# Mechanism research of miR‐34a regulates Axl in non‐small‐cell lung cancer with gefitinib‐acquired resistance

**DOI:** 10.1111/1759-7714.13258

**Published:** 2019-11-27

**Authors:** Ran Xiong, Xiang‐xiang Sun, Han‐ran Wu, Guang‐wen Xu, Gao‐xiang Wang, Xiao‐hui Sun, Mei‐qing Xu, Ming‐ran Xie

**Affiliations:** ^1^ Department of Thoracic Surgery, the First Affiliated Hospital of USTC, Division of Life Sciences and Medicine University of Science and Technology of China Hefei China

**Keywords:** Acquired drug resistance, Gefitinib, miR‐34a, non‐small‐cell lung cancer

## Abstract

**Background:**

To investigate the regulatory mechanism behind miR‐34a‐altered Axl levels in non‐small‐cell lung cancer (NSCLC) with gefitinib‐acquired resistance.

**Methods:**

The expression of miR‐34a, Axl, Gas6 and related downstream signaling proteins in the EGFR mutant NSCLC cell lines were determined by qRT‐PCR and Western blot; PC9‐Gef‐miR‐34a and HCC827‐Gef‐miR‐34a cells were established by transfecting the parent cells with a miR‐34a overexpressing virus, then the expression of Axl, Gas6 and the downstream channel‐related proteins were also compared in PC9‐Gef‐miR‐34a and HCC827‐Gef‐miR‐34a and drug‐resistant strains. The survival rate of the cells were measured by CCK8 assay. A luciferase reporter detected whether Axl was the target of miR‐34a. Finally, a tumor‐bearing nude mouse model was established to verify the relationship between the expression of miR‐34a, Axl and Gas6 mRNA in vivo.

**Results:**

The expression levels of Axl mRNA and protein, Gas6 mRNA and protein, and related downstream proteins in PC9‐Gef and HCC827‐Gef cell lines were higher than those in PC9 and HCC827 parental cell lines, while the expression of miR‐34a was lower than it was in the parental cell lines (*P* < 0.05). The expression of Axl mRNA and protein, Gas6 mRNA and protein, and related downstream signaling proteins in PC9‐Gef and HCC827‐Gef cell lines was higher than the expression in PC9‐Gef‐miR‐34a and HCC827‐Gef‐miR‐34a cells, which overexpressed miR‐34a (*P* < 0.05).

**Conclusion:**

The miR‐34a regulation of Axl plays an important role in NSCLC‐acquired gefitinib resistance, and their expression is inversely correlated, which suggests that they can be used as prognostic markers or potential therapeutic targets for NSCLC.

## Introduction

The discovery of epidermal growth factor receptor‐tyrosine kinase inhibitors (EGFR‐TKIs), such as gefitinib, significantly improve the clinical efficacy of treatments for patients with EGFR‐mutated advanced NSCLC, improving the patient quality of life and the prognosis.^1^, ^2^ However, acquired drug resistance will occur in most patients after a median of 9 to 13 months of treatment.^3^, ^4^, ^5^ The acquired resistance of EGFR‐TKI not only allows the disease to progress in patients but also becomes the bottleneck restricting the continued use of EGFR‐TKI. Therefore, TKI resistance remains a major problem for the molecular targeted therapy of NSCLC. NSCLC can acquire drug resistance through a secondary mutation of exon 20 of EGFR gene (T790M) and the amplification of c‐MET gene; additionally, Axl has been recently found to correlate with the acquired drug resistance of EGFR‐TKI,^5^, ^6^ but the molecular mechanism of Axl leading to EGFR‐TKI resistance in NSCLC lung cancer cells is not fully understood. Increasing evidence suggests that miRNAs may significantly affect the development and chemoresistance of lung cancer, affecting tumor sensitivity to TKI.^7^, ^8^, ^9^ The function of miR‐34a has been increasingly researched in NSCLC studies. Our previous studies found that miR‐34a expression was significantly lower and Axl was more highly expressed in gefitinib‐resistant cell lines than in controls. In this study, the expression of miR‐34a and Axl in EGFR mutant NSCLC cell lines and gefitinib‐resistant strains, as well as proteins in the related downstream PI3K/AKT, MEK/ERK and JAK/STAT signaling pathways, were compared to further explore the relationship between miR‐34a and gefitinib resistance; further, the analysis was performed to clarify whether miR‐34a is involved in the acquired drug resistance of NSCLC with EGFR mutation through regulation of Axl.

## methods

### Cell lines and culture

The human NSCLC cell lines HCC827 and PC9 were purchased from American Type Culture Collection (ATCC) and cultured in RPMI‐1640 medium with 10% FBS and 100 U/mL penicillin/streptomycin at 37°C in a humid atmosphere with 5% CO_2_. Previously published methods^10^ were used to construct gefitinib‐resistant HCC827‐Gef and PC9‐Gef cells. HCC827 and PC9 cells were transfected with overexpressed Axl lentivirus to establish HCC827‐Axl and PC9‐Axl cells. By using the SuperFect liposome transfection reagent and method, the endogenous siRNA (esiAxl) targeting Axl and empty plasmids were transfected into HCC827‐Gef and PC9‐Gef cells to establish the following cell lines: HCC827‐Gef‐esiAxl, HCC827‐Gef‐esiControl, PC9‐Gef‐esiAxl and PC9‐Gef‐esiControl. HCC827‐Gef and PC9‐Gef cells were transfected with a miR‐34a overexpressing lentivirus to establish HCC827‐Gef‐miR‐34a and PC9‐Gef‐miR‐34a cells, respectively.

### Real‐time reverse transcription polymerase chain reaction (qRT‐PCR)

Total RNA was obtained from cultured cells with TRIzol reagent (Invitrogen, USA) according to the manufacturer's instructions. The concentration of isolated total RNA was measured with a NanoDrop ND‐1000 Spectrophotometer (Agilent, CA, USA). Genomic DNA was eliminated and RT was performed using the PrimeScript RT Reagent kit with gDNA Eraser; qPCR was performed using the SYBR Green PCR kit (both Takara Biotechnology Co., Ltd., Dalian, China) according to the manufacturer's recommendations. The process was conducted using a 7900 Real‐Time PCR System (Applied Biosystems; Thermo Fisher Scientific Inc.), β‐actin was used as an internal reference. The primer sequences are shown in Table 1.

**Table 1:** The primer sequence of qRT‐PCR

	Primer sequence	
Gene	Primer F	Primer R
miR‐34a	TGGCAGTGTCTTAGCTGGTT	ATGTGCAGCACTTCTAGGGC
Axl	AGCACACGCGTAAACAACAC	GTTATGGGCTTCGCAGGAGA
Gas6	CAGGACCTCATGGGCAACTT	TTCTCCTGGCTGCATTCGTT
Actin	GCCCTGAGGCTCTCTTCCA	GCGGATGTCGACGTCACA
Actin	GACAGGATGCAGAAGGAGATTACT	TGATCCACATCTGCTGGAAGGT

### Western blot analysis

NSCLC cells were collected, total protein was extracted, and gel electrophoresis was performed to separate proteins by polyacrylic acid amine. Proteins were then transferred to a PVDF membrane, incubated in protein transferring solution at 37°C for 1 hour, washed with PBST, and incubated with primary antibodies (diluted 1:5000, according to manufacturer's instructions) at 4°C with oscillation overnight. The membrane was then washed three times with TBST, secondary antibodies were added for a1 hour incubation, and washing with TBST three times was again carried out, each time for 5 minutes. The ECL method was used at room temperature to develop the images, and the results were recorded. The expression level of each target protein was expressed as the ratio of the target protein band to the internal reference band.

### Luciferase reporter assay

A liposome‐mediated transfection method was used. The experimental groups were pmirGLO+ miR‐NC + pRL‐tk, pmirGLO+ miR‐34a + pRL‐tk, pmirGLO‐Axl 3′ UTR+ miR‐NC + pRL‐tk, and pmirGLO‐Axl 3′ UTR+ miR‐34a + pRL‐tk. Three replicate experiments were performed for each group. PmirGLO was the negative control for pmirGLO‐Axl 3′ UTR, miR‐NC was the negative control for miR‐34a, which was transfected at the same time, and pRL‐tk (sea kidney luciferase) was used as an internal control. Specific transfection methods were in accordance with the Lipofectamine 2000 reagent instructions. Luciferase activity was detected by the dual‐reporter assay kit from Promega, and the instrument for measuring the chemiluminescence resulting from the assay was a 30IOC chemiluminescence analyzer.

### Immunofluorescence analysis

Stable cells were seeded on a BD Falcon 8‐well culture slide and incubated with primary antibodies, rabbit E‐cadherin and vimentin, and were then incubated with Alexa Fluor 594 goat anti‐rabbit IgG. The culture slides were counterstained with Hoechst 33342 and imaged with a confocal laser‐scanning microscope. Data were processed with Adobe Photoshop 7.0 software for analysis.

### Xenograft studies

A total of 20 BALB/c nude mice (male, 5–6 weeks old, weighing 20‐25g in weight), purchased from Shanghai Laboratory Animal Center, were selected and divided into two groups of 10 mice. One group was inoculated with a 200 μL suspension (1 × 10^8^/mL) of HCC827‐Gef cells on the posterior flank skin, and the other group was inoculated with PC9‐Gef cells. Tumor growth in the mice was assessed three times a week after inoculation. Tumors were measured with vernier calipers three times a week after inoculation, with volumes derived as follows: Volume = 0.5 × Length × (Width)^2^.

### Statistical analysis

Data were analyzed using SPSS statistical software, version 17.0 (SPSS, Inc., Chicago, IL, USA). The comparison of the expression levels of miR‐34a, Axl, Gas6 and downstream pathway‐related proteins between the two groups was conducted using a *t*‐test with two independent samples. The test level was set at a = 0.05, and *P* < 0.05 indicated that the difference was statistically significant.

## Results

### Cell survival test for HCC827, PC9, HCC827‐Gef and PC9‐Gef

**Figure 1:**
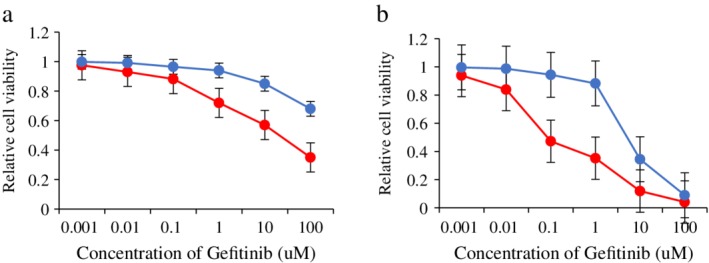
Cell survival test of HCC827, PC9, HCC827‐Gef and PC9‐Gef. (**a**) The sensitivity of HCC827 and HCC827‐Gef cells to gefitinib was examined by CCK8 assay. (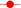) HCC827 and (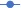) HCC827‐Gef. (**b**) The sensitivity of PC9 and PC9‐Gef cells to gefitinib was examined by CCK8 assay. (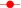) PC9 and (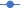) PC9‐Gef.

When in the logarithmic growth phase, HCC827, HCC827‐Gef, PC9 and PC9‐Gef cells were treated with gefitinib at different concentrations (0.001, 0.01, 0.1, 1, 10, and 100 μmol/L). After 48 hours, a CCK8 assay was used to test the cell survival rate. Compared with parental cells HCC827 and PC9 cells treated with gefitinib at different concentrations, the survival rate of HCC827‐Gef and PC9‐Gef cells was higher and their sensitivity to gefitinib was lower (*P* < 0.05), as shown in Figure 1.

### Expression of miR‐34a, Axl and Gas6 in HCC827, HCC827‐Gef, PC9 and PC9‐Gef cells

**Figure 2:**
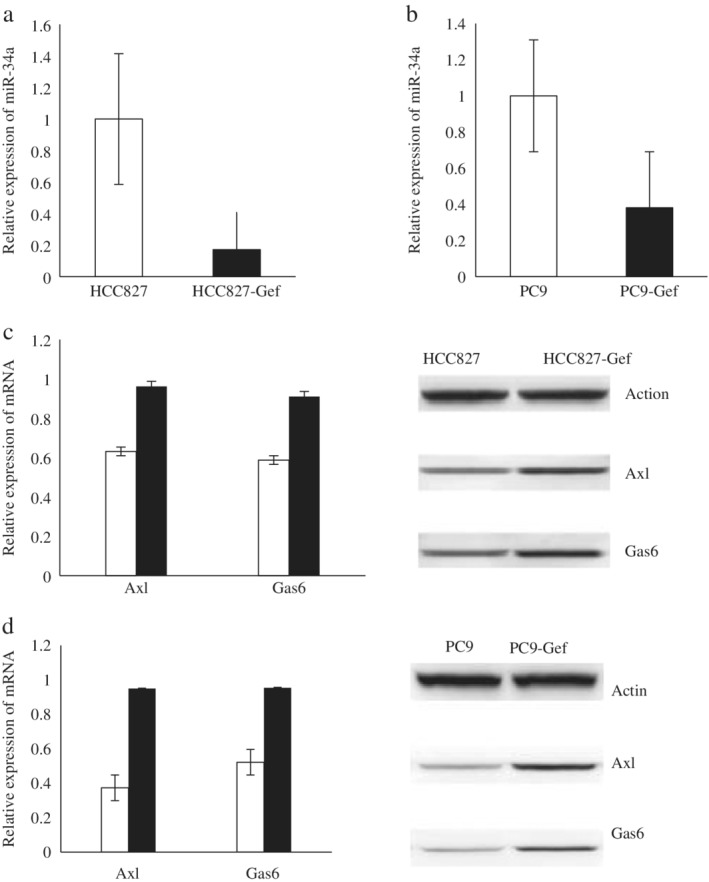
The expression of miR‐34a, Axl and Gas6 in HCC827, HCC827‐Gef, PC9 and PC9‐Gef. (**a**) The relative expression of miR‐34a in HCC827 and HCC827‐Gef cells by RT‐qPCR analysis. (**b**) The relative expression of miR‐34a in PC9 and PC9‐Gef cells by RT‐qPCR analysis. (**c**) The expression of Axl, Gas6 mRNA and protein in HCC827 and HCC827‐Gef cells by RT‐qPCR and western blot analysis. (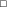) HCC827 and (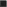) HCC827‐Gef. (**d**) The expression of Axl, Gas6 mRNA and protein in PC9 and PC9‐Gef cells by RT‐qPCR and western blot analysis. (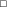) PC9 and (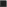) PC9‐Gef.

Using qRT‐PCR and Western blot analysis, it was found that the expression levels of Axl and Gas6 mRNA and protein in HCC827‐Gef cells were significantly higher than those in HCC827 cells, and the expression levels of Axl and Gas6 mRNA and protein in PC9‐Gef cells were also significantly higher than those in PC9 cells. The expression of miR‐34a in drug‐resistant strains was lower than that in parental cells. The differences in gene expression between the drug‐resistant strains and their parent strains were statistically significant (*P* < 0.05), as shown in Figure 2.

### Expression of downstream signaling proteins and sensitivity of each group to gefitinib compared after upregulating and downregulating Axl

**Figure 3:**
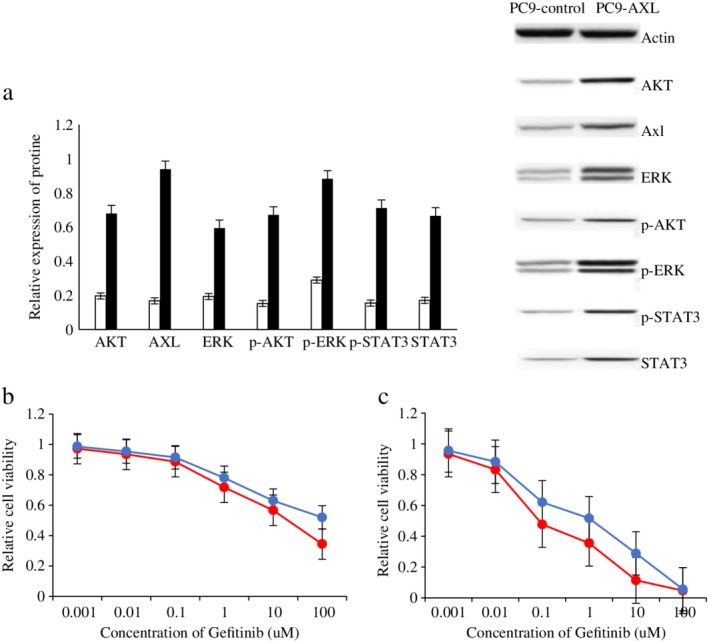
The expression of downstream channel related proteins and the sensitivity to gefitinib in each group were compared after upregulating Axl. (**a**) The expression of Axl, Gas6 and downstream channel related proteins in HCC827‐control and HCC827‐Axl cells by western blot analysis. (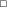) HCC827‐control and (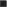) HCC827‐AXL. (**b**) The expression of Axl, Gas6 and downstream channel related proteins in PC9‐control and PC9‐Axl cells by western blot analysis. (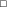) PC9‐control and (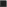) PC9‐AXL. (**c**) The sensitivity of HCC827‐control and HCC827‐Axl cells to gefitinib was examined by CCK8 assay. (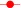) HCC827 and (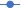) HCC827‐AXL. (**d**) The sensitivity of PC9‐control and PC9‐Axl cells to gefitinib was examined by CCK8 assay. (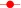) PC9 and (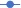) PC9‐AXL.

**Figure 4:**
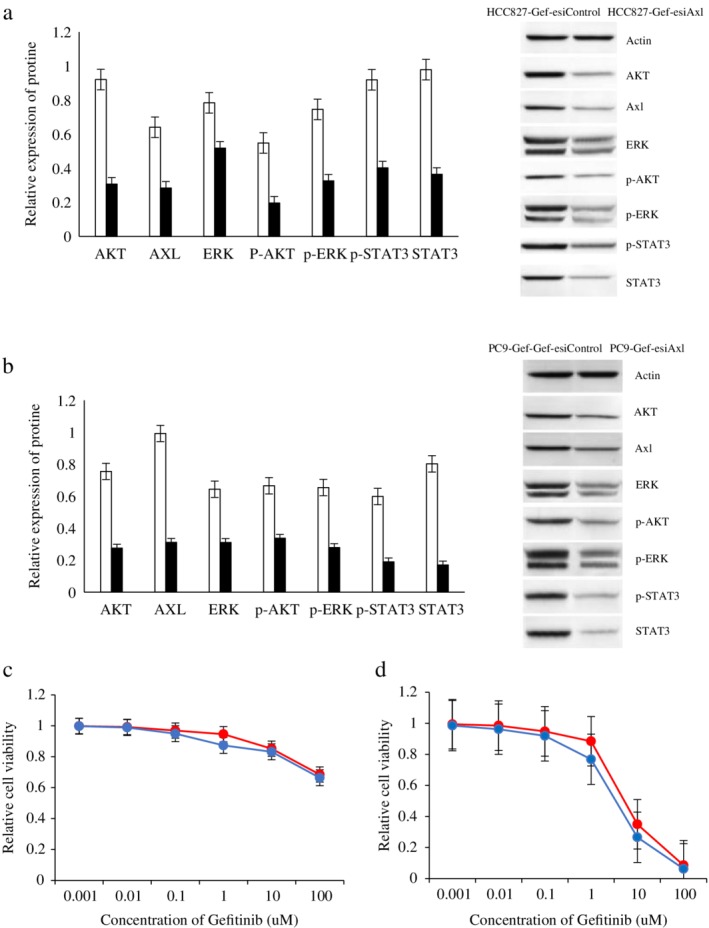
The expression of downstream channel related proteins and the sensitivity to gefitinib in each group were compared after downregulating Axl. (**a**) The expression of Axl, Gas6 and downstream channel related proteins in HCC827‐Gef‐esiControl and HCC827‐Gef‐esiAxl cells by western blot analysis. (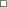) HCC827‐Gef and (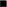) HCC827‐Gef‐siRNA. (**b**) The expression of Axl, Gas6 and downstream channel related proteins in PC9‐Gef‐esiControl and PC9‐Gef‐esiAxl cells by western blot analysis. (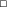) PC9‐Gef and (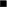) PC9‐Gef‐siRNA. (**c**) The sensitivity of HCC827‐Gef‐esiControl and HCC827‐Gef‐esiAxl cells to gefitinib was examined by CCK8 assay. (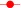) HCC827‐Gef and (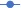) HCC827‐Gef‐siRNA. (**d**) The sensitivity of PC9‐Gef‐esiControl and PC9‐Gef‐esiAxl cells to gefitinib was examined by CCK8 assay. (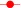) PC9‐Gef and (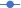) PC9‐Gef‐siRNA.

HCC827 and PC9 cells were transfected with an Axl overexpressing lentivirus to obtain HCC827‐Axl and PC9‐Axl cells, and HCC827‐Gef‐esiAxl and PC9‐Gef‐esiAxl cells were obtained by using the SuperFect liposome transfection reagent and method. The expression of downstream signaling proteins between the high and low Axl‐expressing cells and the parental cells was compared, and the changes in sensitivity to gefitinib were also compared. The results showed that the expression levels of AKT, p‐AKT, ERK, p‐ERK, STAT3 and p‐STAT3 in HCC827‐Axl and PC9‐Axl cells were significantly higher than those in HCC827‐control and PC9‐control cells; further, the sensitivity of HCC827‐Axl and PC9‐Axl cells to gefitinib was decreased after overexpressing Axl, and the differences were significantly different (*P* < 0.05). The expression levels of AKT, p‐AKT, ERK, p‐ERK, STAT3 and p‐STAT3 in HCC827‐Gef‐esiAxl and PC9‐Gef‐esiAxl cells were significantly lower than those in the HCC827‐Gef‐esiControl and PC9‐Gef‐esiControl cells, and the sensitivity of HCC827‐Gef‐esiAxl and PC9‐Gef‐esiAxl cells to gefitinib was significantly higher than that in the control cells (*P* < 0.05). The results are shown in Figures 3 and 4.

### Detection of expression of Axl and Gas6 in HCC827‐Gef‐miR‐34a, HCC827‐Gef‐Control, PC9‐Gef‐miR‐34a and PC9‐Gef‐Control cells; survival rates of each group of cells after gefitinib treatment was detected by CCK8

**Figure 5:**
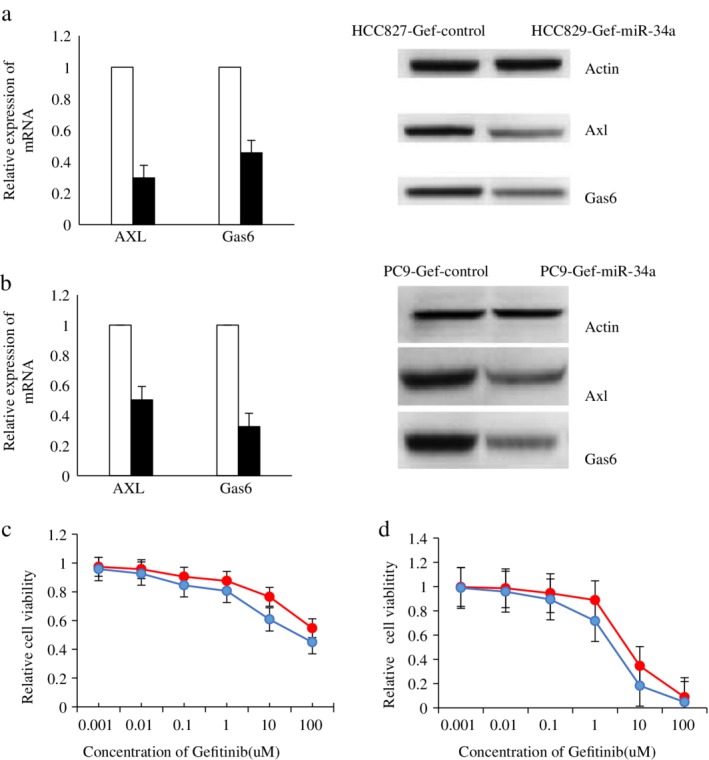
The expression of Axl, Gas6 and the sensitivity to gefitinib were compared in HCC827‐Gef‐Control, HCC827‐Gef‐miR‐34a, PC9‐Gef‐Control and PC9‐Gef‐miR‐34a cells. (**a**) The expression of Axl, Gas6 mRNA and protein in HCC827‐Gef‐Control and HCC827‐Gef‐miR‐34a cells by RT‐qPCR and western blot analysis. (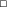) HCC827‐Gef‐Control and (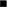) HCC827‐Gef‐miR‐34a. (**b**) The expression of Axl, Gas6 mRNA and protein in PC9‐Gef‐Control and PC9‐Gef‐miR‐34a cells by RT‐qPCR and western blot analysis. (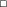) PC9‐Gef‐Control and (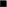) PC9‐Gef‐miR‐34a. (**c**) The sensitivity of HCC827‐Gef‐Control and HCC827‐Gef‐miR‐34a cells to gefitinib was examined by CCK8 assay. (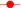) HCC827‐Gef‐Control and (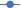) HCC827‐Gef‐miR‐34a. (**d**) The sensitivity of PC9‐Gef‐Control and PC9‐Gef‐miR‐34a cells to gefitinib was examined by CCK8 assay. (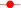) PC9‐Gef‐Control and (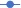) PC9‐Gef‐miR‐34a.

HCC827‐Gef and PC9‐Gef cells were transfected with the miR‐34a overexpressing lentivirus to establish HCC827‐Gef‐miR‐34a and PC9‐Gef‐miR‐34a cells. The expression of Axl and Gas6 was compared, and the results showed that the expression levels of Axl and Gas6 in HCC827‐Gef‐miR‐34a and PC9‐Gef‐miR‐34a cells were significantly lower than those in HCC827‐Gef‐Control and PC9‐Gef‐Control cells (*P* < 0.05). When HCC827‐Gef‐miR‐34a, HCC827‐Gef‐Control, PC9‐Gef‐miR‐34a and PC9‐Gef‐Control cells were in the logarithmic growth phase, they were treated with gefitinib at different concentrations (0.001, 0.01, 0.1, 1, 10, and 100 μmol/L). After 48 hours, CCK8 assays were used to test the cell survival rate. The results showed that the sensitivity of HCC827‐Gef‐miR‐34a and PC9‐Gef‐miR‐34a cells to gefitinib was higher after transfection with the miR‐34a overexpressing lentivirus compared to the control, and the differences were significant (*P* < 0.05). All results are shown in Figure 5.

### Axl is target of miR‐34a

**Figure 6:**
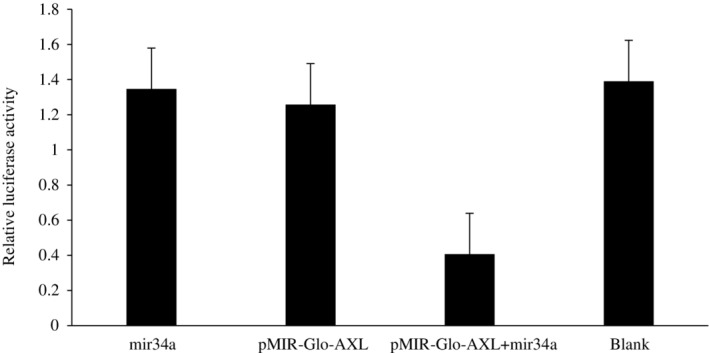
Luciferase reporter gene (miR‐34a targets Axl).

A luciferase reporter gene was used, and the ratio of the pmir‐GLO‐Axl + miR‐34a group was significantly reduced compared to the control group. The difference was statistically significant (*P* < 0.05), indicating that Axl was targeted by miR‐34a. The results are shown in Figure 6.

### Nude mouse model established to verify in vitro and in vivo experimental results

**Figure 7:**
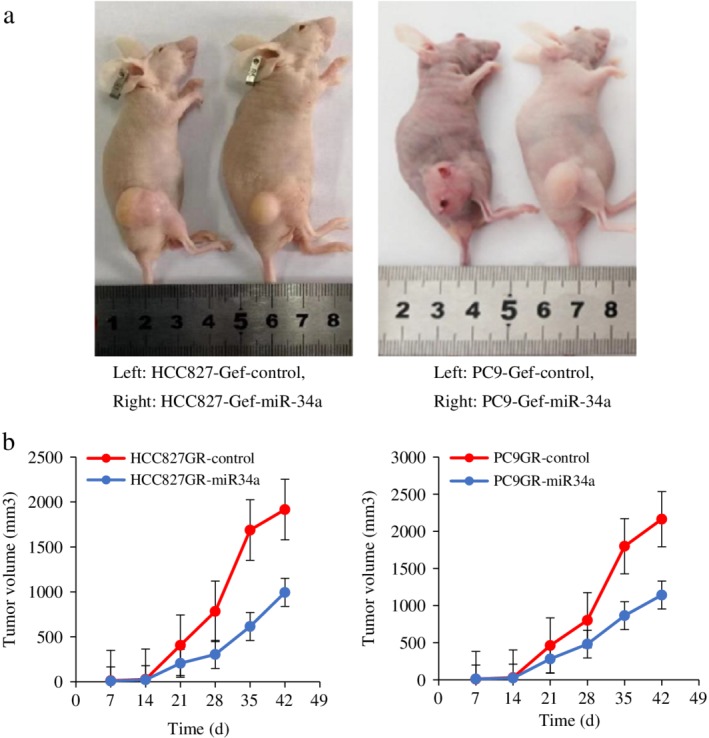
The nude mice model was established to verify the in vitro and in vivo experimental results. (**a**) Representative image for tumor growth is shown. (**b**) Determination of the tumor growth. The tumor volume was calculated every week after injection.

A total of 10 days after inoculation, half of the nude mice in each group were injected with the miR‐34a overexpressing virus via the tail vein, and all nude mice were administered gefitinib via gavage, five days per week for four to six weeks. Tumor volume changes in each group were measured at 0, 7, 14, 21, 28, 35 and 42 days after injection (seven time points). Data are mean ± S.D of three independent experiments. Compared with the control group, the tumor size in miR‐34a overexpressing nude mice were significantly reduced (*P* < 0.05), and the results are shown in Figure 7. The nude mice were killed 42 days after injection.Axl, Gas6, AKT, p‐AKT, ERK1/2, p‐ERK1/2, STAT3 and p‐STAT3 were detected by immunohistochemistry, compared with the control group, the expression of related proteins in downstream pathways was significantly reduced in miR‐34a overexpressing nude mice (*P* < 0.05), as shown in Figure S1.

## Discussion

Although the use of TKI has provided a beneficial prognosis for some patients who have advanced non‐small‐cell lung cancer with EGFR mutations, it has been found in clinical practice that most treated patients have different degrees of drug resistance after 9 to 13 months.^3^, ^4^, ^5^ The mechanism of acquired drug resistance is not completely clear, and currently, the widely recognized mechanisms include a secondary mutation of exon 20 in the EGFR gene (T790M) and amplification of the MET gene. The latest research showed that Axl was used to control the acquired TKI resistance of NSCLC.^5^, ^6^, ^11^, ^12^, ^13^, ^14^ Anexelekto (Axl) is a member of the receptor tyrosine kinase subfamily. Binding to growth arrest specific protein 6 (Gas6) can activate its tyrosine kinase activity and activate downstream signal transduction pathways, where it participates in cell adhesion, proliferation, apoptosis and other processes. Although there have been some studies that have examined the mechanism of how Axl induces TKI resistance in NSCLC, the specific mechanism is still unclear.

In this study, we mainly focused on Axl regulation by miR‐34a and the expression of downstream signaling proteins. A miRNA can target multiple mRNAs to regulate cell proliferation, differentiation, apoptosis and other biological function.^15^, ^16^, ^17^ It was found that in gefitinib‐resistant cell lines, there was an inverse relationship between the expression of miR‐34a, Axl and Gas6 in parental cells and drug‐resistant strain cells, and there was an isotropic relationship between Axl, Gas6 and downstream PI3K/AKT, MEK/ERK and JAK/STAT related proteins. In the cell lines with gefitinib resistance, when we overexpressed miR‐34a with a lentivirus, we found that Axl and Gas6 expression in drug‐resistant strains was significantly lower than that in the control group, and the sensitivity of the resistant strains to gefitinib increased. When Axl was downregulated through esiRNA treatment, we found that the expression of downstream signaling proteins was significantly reduced compared with that in the control group, and the sensitivity of the cells to gefitinib increased. Our data show that miR‐34a may regulate gefitinib resistance by downregulating Axl and the expression of downstream signaling proteins, and the results are consistent with the previous research.^18^, ^19^

To date, related studies have found abnormal expression of miR‐34a in a variety of tumors and cell lines, including neuroblastoma, lung cancer, and melanoma cell lines.^20^, ^21^, ^22^, ^23^ We found that Axl was a potential target of miR‐34a using Target Scan bioinformatics software, and further double luciferase reporter gene assays revealed that Axl was targeted by miR‐34a. We have confirmed the inverse relationship between miR‐34a and Axl through cell experiments, then established a nude mouse model. After the tail vein of tumor‐carrying nude mice was injected with a lentivirus expressing miR‐34a, it was found that the tumor size were significantly reduced compared with the control group; further, the expression of related proteins in downstream pathways was significantly reduced, as measured by immunohistochemistry. The results showed that the upregulation of miR‐34a in vivo could further increase the sensitivity of gefitinib‐resistant NSCLC cells to gefitinib. Thus, miR‐34a could be used as a biomarker for NSCLC prognosis and gefitinib efficacy.

There are some limitations to our study. First, due to time constraints, no clinical specimens were included which would have enabled the study of patient survival and prognosis. Second, the number of cases was relatively small due to objective reasons. Nevertheless, the current results are the first to suggest that miR‐34a plays an important role in inhibiting lung cancer cell growth by inhibiting Axl. Although we speculated that miR‐34a could inhibit lung cancer by targeting Axl, there was only one miRNA and one mRNA analyzed in this study, and there may be other miR‐34a targets or other miRNAs affecting the growth of lung cancer tumors. Therefore, further research is needed to confirm this hypothesis.

In conclusion, we investigated whether miR‐34a regulates Axl in gefitinib‐resistant cell lines. We found that dysregulated miR‐34a could change tumorigenesis and gefitinib resistance in NSCLC through Axl by affecting the related downstream signaling pathways of PI3K/AKT, MEK/ERK and JAK/STAT in vitro and in vivo. Therefore, miR‐34a may serve not only as a therapeutic target but also as a prognostic biomarker for NSCLC.

## Disclosure

The authors declare no conflicts of interest.

## Supporting information


**Figure S1** Axl was detected in HCC827‐Gef‐control, HCC827‐Gef‐miR‐34a, PC9‐Gef‐control and PC9‐Gef‐miR‐34a mice by immunohistochemistry(400X).
**Figure S2** Gas6 was detected in HCC827‐Gef‐control, HCC827‐Gef‐miR‐34a, PC9‐Gef‐control and PC9‐Gef‐miR‐34a mice by immunohistochemistry(400X).
**Figure S3** AKT was detected in HCC827‐Gef‐control, HCC827‐Gef‐miR‐34a, PC9‐Gef‐control and PC9‐Gef‐miR‐34a mice by immunohistochemistry(400X).
**Figure S4** p‐AKT was detected in HCC827‐Gef‐control, HCC827‐Gef‐miR‐34a, PC9‐Gef‐control and PC9‐Gef‐miR‐34a mice by immunohistochemistry(400X).
**Figure S5** ERK was detected in HCC827‐Gef‐control, HCC827‐Gef‐miR‐34a, PC9‐Gef‐control and PC9‐Gef‐miR‐34a mice by immunohistochemistry(400X).
**Figure S6** p‐ERK was detected in HCC827‐Gef‐control, HCC827‐Gef‐miR‐34a, PC9‐Gef‐control and PC9‐Gef‐miR‐34a mice by immunohistochemistry(400X).
**Figure S7** STAT3 was detected in HCC827‐Gef‐control, HCC827‐Gef‐miR‐34a, PC9‐Gef‐control and PC9‐Gef‐miR‐34a mice by immunohistochemistry(400X).
**Figure S8** p‐STAT3 was detected in HCC827‐Gef‐control, HCC827‐Gef‐miR‐34a, PC9‐Gef‐control and PC9‐Gef‐miR‐34a mice by immunohistochemistry(400X).

## References

[1] Zhou C , Wu YL , Chen G *et al* Erlotinib versus chemotherapy as first‐line treatment for patients with advanced EGFR mutation‐positive non‐small‐cell lung cancer (OPTIMAL, CTONG‐0802): A multicentre, open‐label, randomised, phase 3 study. Lancet Oncol 2011; 12: 735–42. 21783417 10.1016/S1470-2045(11)70184-X

[2] Ricciuti B , Mecca C , Cenci M *et al* miRNAs and resistance to EGFR‐TKIs in EGFR‐mutant non‐small cell lung cancer: Beyond 'traditional mechanisms' of resistance. Ecancermedicalscience 2015; 2 (9): 569. 10.3332/ecancer.2015.569 PMC4583238 26435742

[3] Maemondo M , Inoue A , Kobayashi K *et al* Gefitinib or chemotherapy for non‐small‐cell lung cancer with mutated EGFR. N Engl J Med 2010; 362: 2380–8. 20573926 10.1056/NEJMoa0909530

[4] Mitsudomi T , Morita S , Yatabe Y *et al* Gefitinib versus cisplatin plus docetaxel in patients with non‐small‐cell lung cancer harboring mutations of the epidermal growth factor receptor (WJTOG3405): An open label, randomized phase 3 trial. Lancet Oncol 2010; 11: 121–8. 20022809 10.1016/S1470-2045(09)70364-X

[5] Byers LA , Diao L , Wang J *et al* An epithelial‐mesenchymal transition gene signature predicts resistance to EGFR and PI3K inhibitors and identifes Axl as a therapeutic target for overcoming EGFR inhibitor resistance. Clin Cancer Res 2013; 19: 279–90. 23091115 10.1158/1078-0432.CCR-12-1558 PMC3567921

[6] Wang Y , Xia H , Zhuang Z , Miao L , Chen X , Cai H . Axl‐altered microRNAs regulate tumorigenicity and gefitinib resistance in lung cancer. Cell Death Dis 2014; 5 (5): e1227. 24832599 10.1038/cddis.2014.186 PMC4047906

[7] Pan B , Feng B , Chen Y *et al* MiR‐200b regulates autophagy associated with chemoresistance in human lung adenocarcinoma. Oncotarget 2015; 6: 32805–20. 26416454 10.18632/oncotarget.5352 PMC4741731

[8] Deng Z , Rong Y , Teng Y *et al* Exosomes miR‐126a released from MDSC induced by DOX treatment promotes lung metastasis. Oncogene 2017; 36 (5): 639–51. 27345402 10.1038/onc.2016.229 PMC5419051

[9] Ye Z , Yin S , Su Z *et al* Downregulation of miR‐101 contributes to epithelial mesenchymal transition in cisplatin resistance of NSCLC cells by targeting ROCK2. Oncotarget 2016; 7: 37524–35. 27229528 10.18632/oncotarget.6852 PMC5122329

[10] Wang YS , Wang YH , Xia HP et al. MicroRNA‐214 regulates the acquired resistance to gefitinib via the PTEN/AKT pathway in EGFR‐mutant cell lines. Asian Pac J Cancer Prev 2012; 13 (1): 255–60. 22502680 10.7314/apjcp.2012.13.1.255

[11] Wu X , Liu X , Koul S , Lee CY , Zhang Z , Halmos B . AXL kinase as a novel target for cancer therapy. Oncotarget 2014; 5 (20): 9546–63. 25337673 10.18632/oncotarget.2542 PMC4259419

[12] Bae SY , Hong JY , Lee HJ , Park HJ , Lee SK . Targeting the degradation of AXL receptor tyrosine kinase to overcome resistance in gefitinib‐resistant non‐small cell lung cancer. Oncotarget 2015; 6 (12): 10146–60. 25760142 10.18632/oncotarget.3380 PMC4496346

[13] Kim D , Bach DH , Fan YH *et al* AXL degradation in combination with EGFR‐TKI can delay and overcome acquired resistance in human non‐small cell lung cancer cells. Cell Death Dis 2019; 10 (5): 361. 31043587 10.1038/s41419-019-1601-6 PMC6494839

[14] Nonagase Y , Takeda M , Azuma K *et al* Tumor tissue and plasma levels of AXL and GAS6 before and after tyrosine kinase inhibitor treatment in EGFR‐mutated non‐small cell lung cancer. Thorac Cancer 2019; 10: 1928–35. 31419057 10.1111/1759-7714.13166 PMC6775020

[15] Ning ZQ , Lu HL , Chen C *et al* MicroRNA‐30e reduces cell growth and enhances drug sensitivity to gefitinib in lung carcinoma. Oncotarget 2017; 8 (3): 4572–81. 27992364 10.18632/oncotarget.13944 PMC5354855

[16] Garofalo M , Jeon Y , Nuovo GJ *et al* MiR‐34a/c‐dependent PDGFR‐α/β Downregulation inhibits tumorigenesis and enhances TRAIL‐induced apoptosis in lung cancer. PLOS One 2013; 8 (6): e67581. 23805317 10.1371/journal.pone.0067581 PMC3689725

[17] Yang X , Zhang Q , Zhang M *et al* Serum microRNA signature is capable of early diagnosis for non‐small cell lung cancer. Int J Biol Sci 2019; 15 (8): 1712–22. 31360113 10.7150/ijbs.33986 PMC6643220

[18] Cho CY , Huang JS , Shiah SG *et al* Negative feedback regulation of AXL by miR‐34a modulates apoptosis in lung cancer cells. RNA 2016; 22 (2): 303–15. 26667302 10.1261/rna.052571.115 PMC4712679

[19] Mudduluru G , Ceppi P , Kumarswamy R , Scagliotti GV , Papotti M , Allgayer H . Regulation of Axl receptor tyrosine kinase expression by miR‐34a and miR‐199a/b in solid cancer. Oncogene 2011; 30 (25): 2888–99. 21317930 10.1038/onc.2011.13

[20] Cheng X , Xu Q , Zhang Y *et al* miR‐34a inhibits progression of neuroblastoma by targeting autophagy‐related gene 5. Eur J Pharmacol 2019; 850: 53–63. 30716314 10.1016/j.ejphar.2019.01.071

[21] Kim JS , Kim EJ , Lee S *et al* MiR‐34a and miR‐34b/c have distinct effects on the suppression of lung adenocarcinomas. Exp Mol Med 2019; 51 (1): 9. 30700696 10.1038/s12276-018-0203-1 PMC6353903

[22] Wang F , Li Z , Xu L *et al* LIMD2 targeted by miR‐34a promotes the proliferation and invasion of non‐small cell lung cancer cells. Mol Med Rep 2018; 18 (5): 4760–6. 30221696 10.3892/mmr.2018.9464

[23] Liu R , Xie H , Luo C *et al* Identification of FLOT2 as a novel target for microRNA‐34a in melanoma. J Cancer Res Clin Oncol 2015; 141 (6): 993–1006. 25403318 10.1007/s00432-014-1874-1 PMC11824034

