# Supplementary material for: Mechanism research of miR‐34a regulates Axl in non‐small‐cell lung cancer with gefitinib‐acquired resistance
Source: Thorac Cancer. 2019 Nov 27;11(1):156–65. doi: 10.1111/1759-7714.13258 (PMC6938762; doi:10.1111/1759-7714.13258)

Figure 1 Axl was detected in HCC827-Gef-control, HCC827-Gef-miR-34a, PC9-Gef-control and PC9-Gef-miR-34a mice by immunohistochemistry（400X）.

HCC827-Gef-control HCC827-Gef-miR-34a


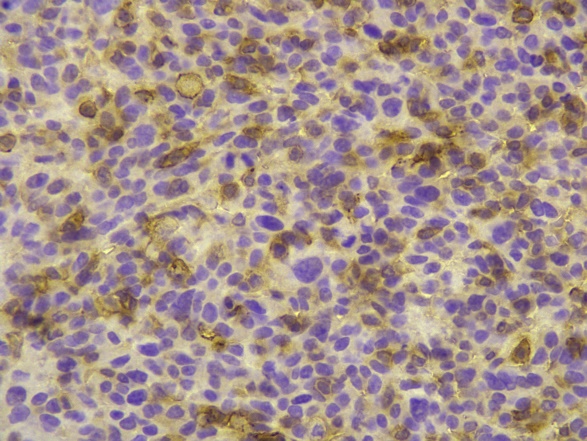

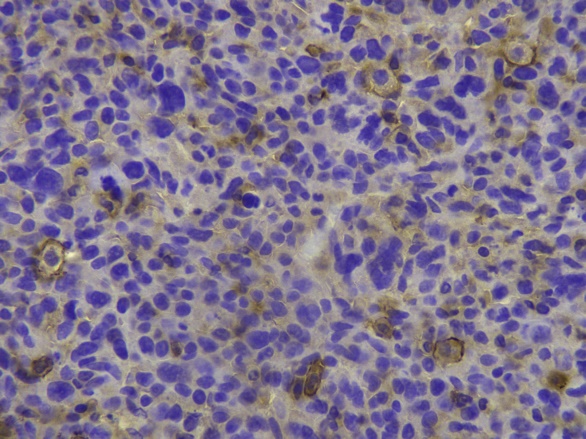


PC9-Gef-control PC9-Gef-miR-34a


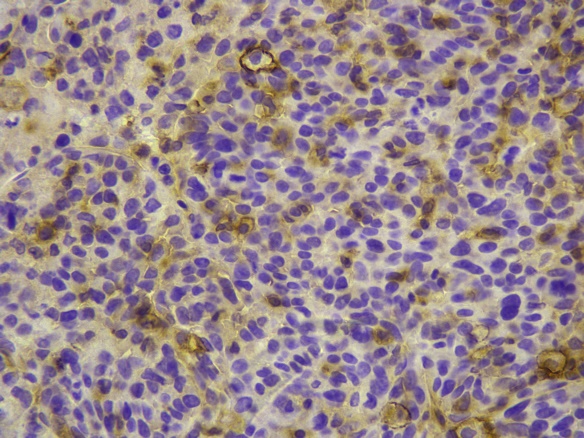

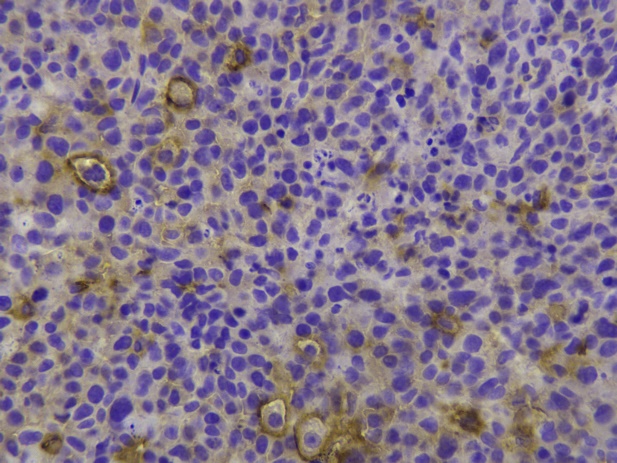


Figure 2 Gas6 was detected in HCC827-Gef-control, HCC827-Gef-miR-34a, PC9-Gef-control and PC9-Gef-miR-34a mice by immunohistochemistry（400X）.

HCC827-Gef-control HCC827-Gef-miR-34a


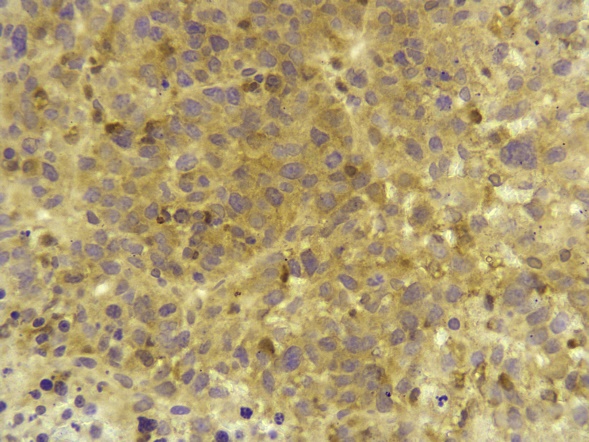

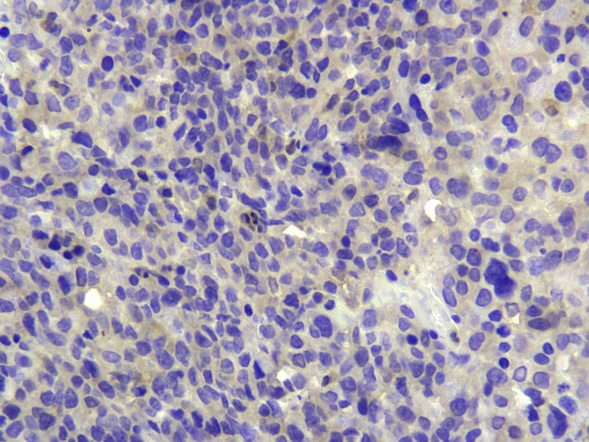


PC9-Gef-control PC9-Gef-miR-34a


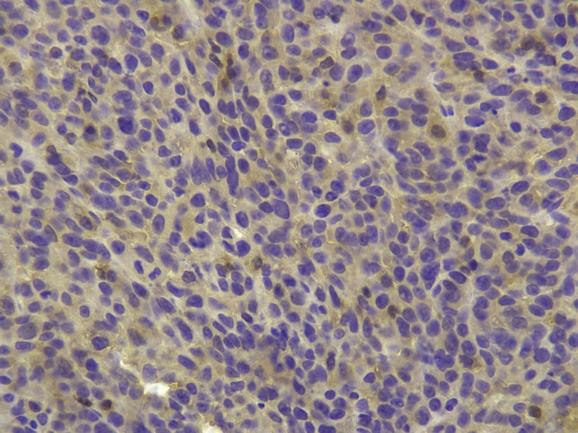

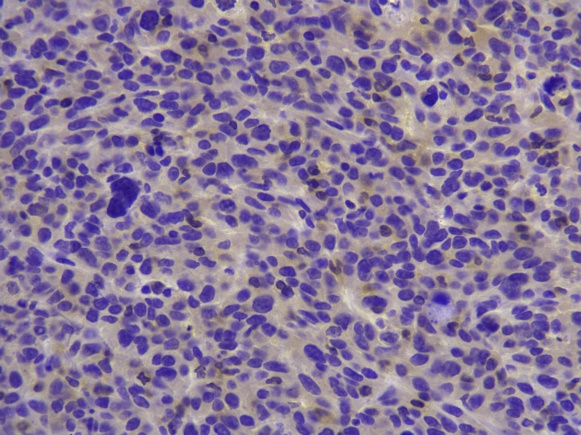


Figure 3 AKT was detected in HCC827-Gef-control, HCC827-Gef-miR-34a, PC9-Gef-control and PC9-Gef-miR-34a mice by immunohistochemistry（400X）.

HCC827-Gef-control HCC827-Gef-miR-34a


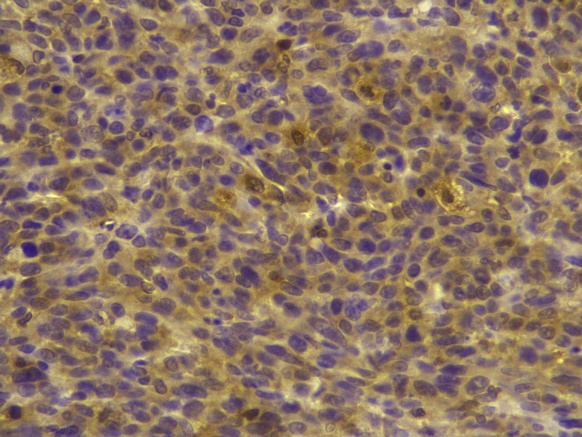

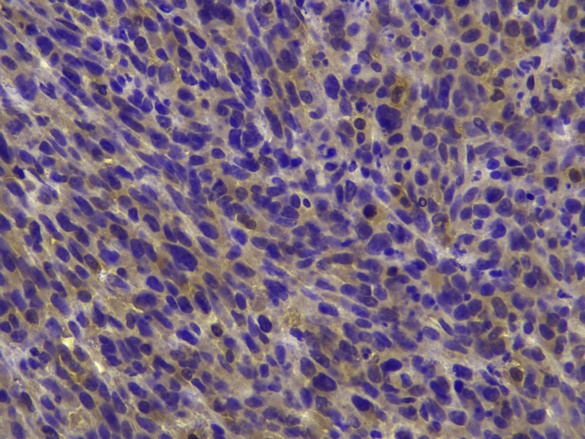


PC9-Gef -control PC9-Gef-miR-34a


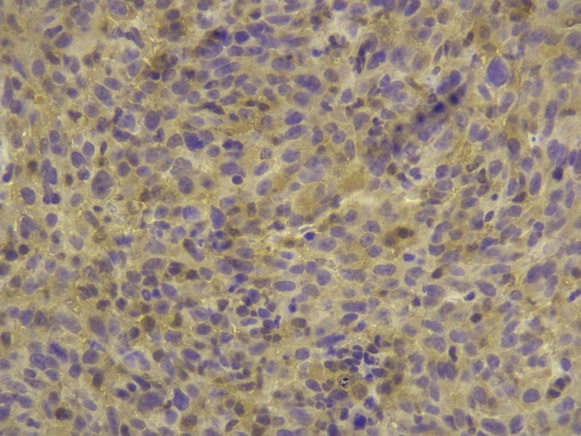

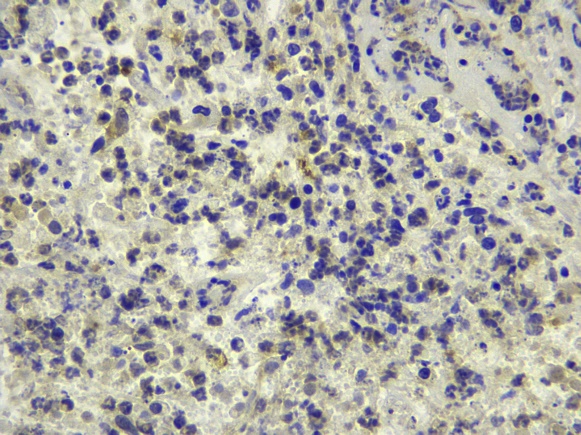


Figure 4 p-AKT was detected in HCC827-Gef-control, HCC827-Gef-miR-34a, PC9-Gef-control and PC9-Gef-miR-34a mice by immunohistochemistry（400X）.

HCC827-Gef-control HCC827-Gef-miR-34a


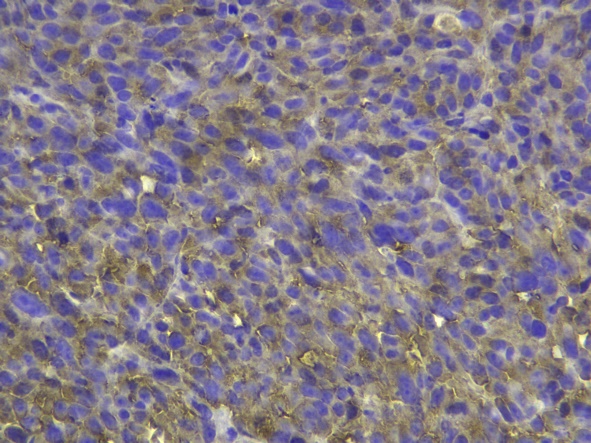

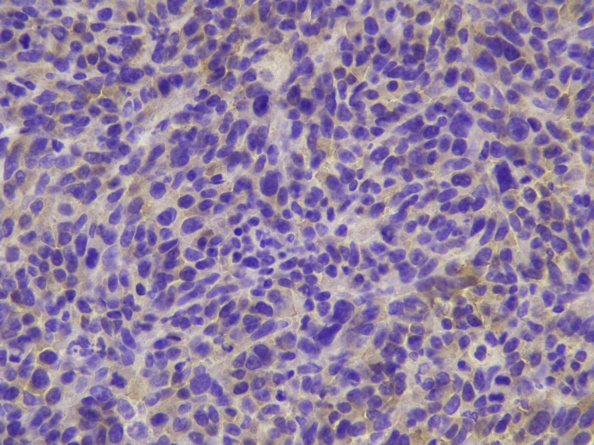


PC9-Gef-control PC9-Gef-miR-34a


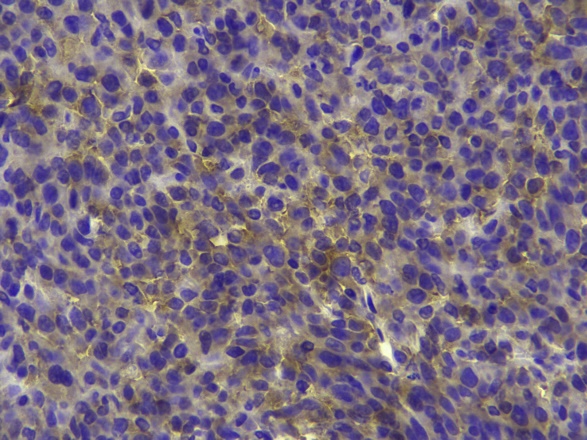

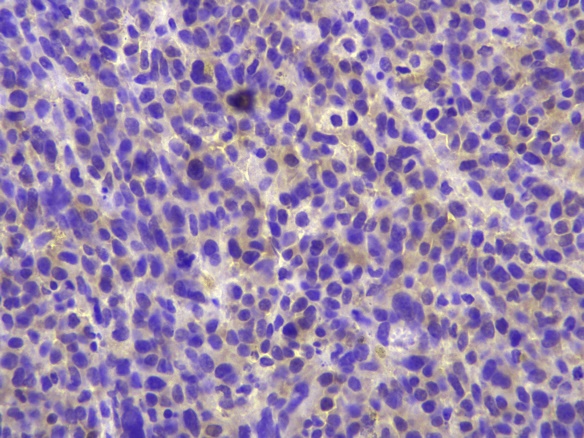


Figure 5 ERK was detected in HCC827-Gef-control, HCC827-Gef-miR-34a, PC9-Gef-control and PC9-Gef-miR-34a mice by immunohistochemistry（400X）.

HCC827-Gef-control HCC827-Gef-miR-34a


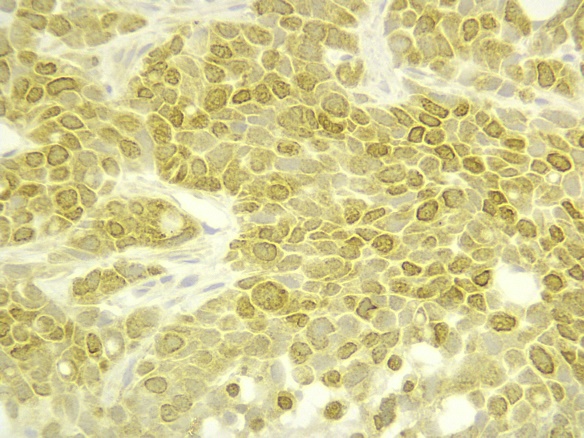

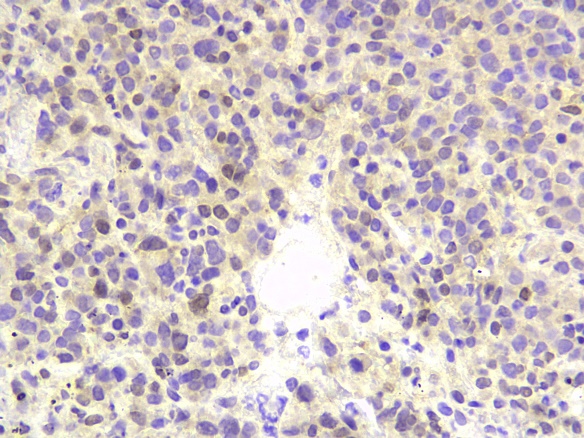


PC9-Gef-control PC9-Gef-miR-34a


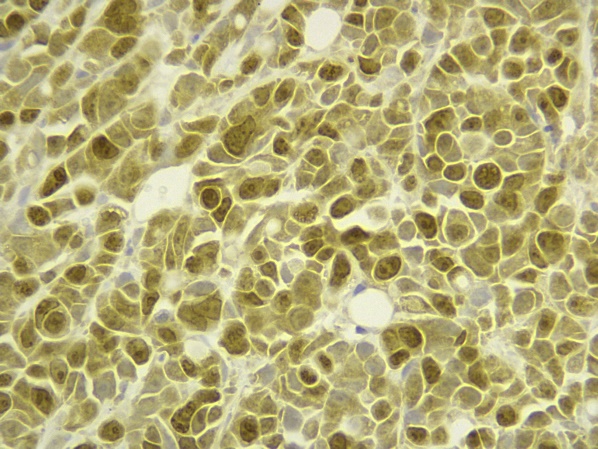

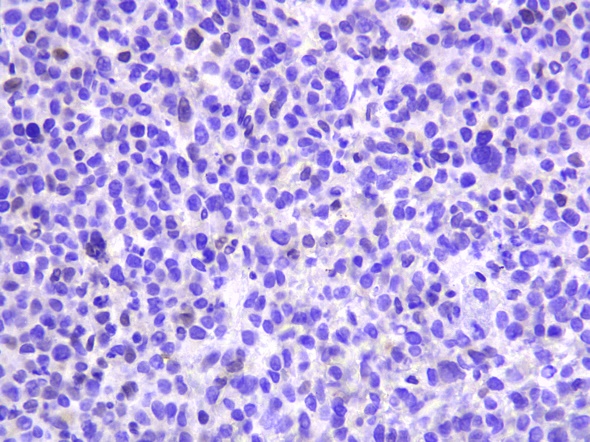


Figure 6 p-ERK was detected in HCC827-Gef-control, HCC827-Gef-miR-34a, PC9-Gef-control and PC9-Gef-miR-34a mice by immunohistochemistry（400X）.

HCC827-Gef-control HCC827-Gef-miR-34a


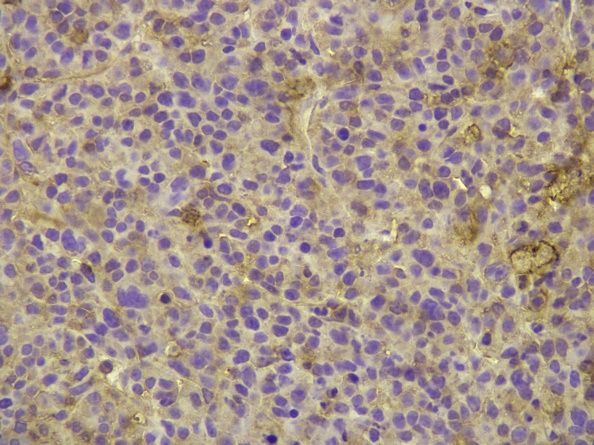

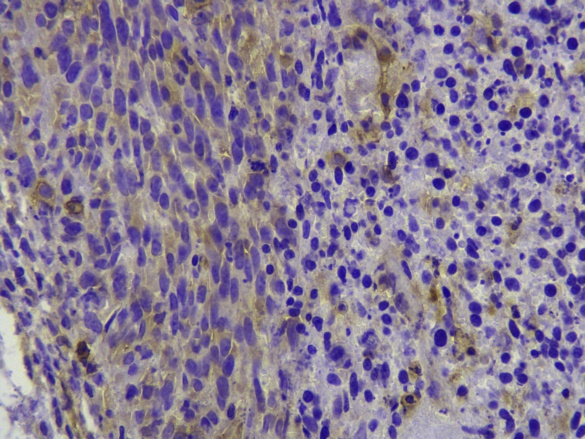


PC9-Gef-control PC9-Gef-miR-34a


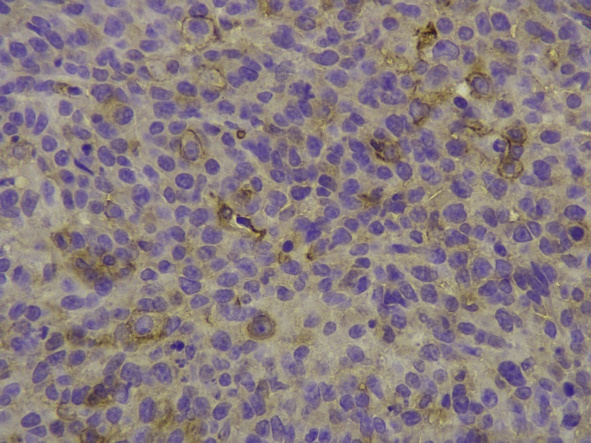

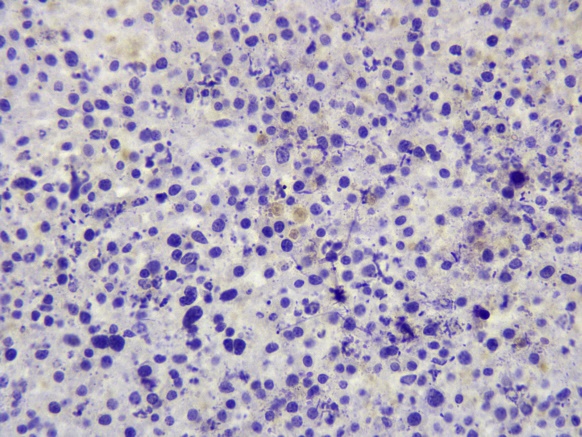


Figure 7 STAT3 was detected in HCC827-Gef-control, HCC827-Gef-miR-34a, PC9-Gef-control and PC9-Gef-miR-34a mice by immunohistochemistry（400X）.

HCC827-Gef-control HCC827-Gef-miR-34a


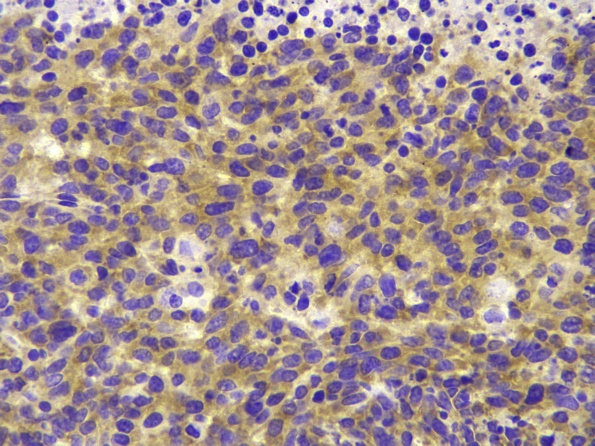

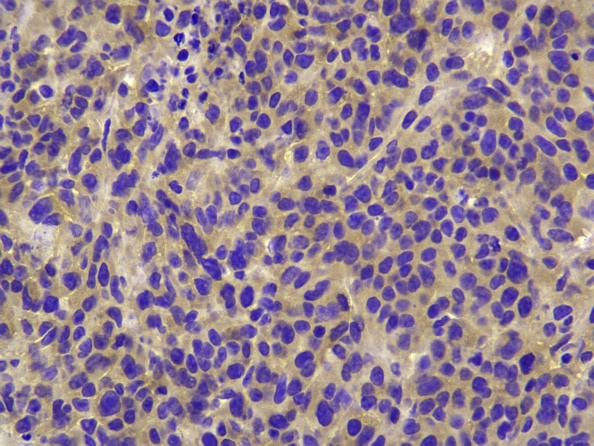


PC9-Gef-control PC9-Gef-miR-34a


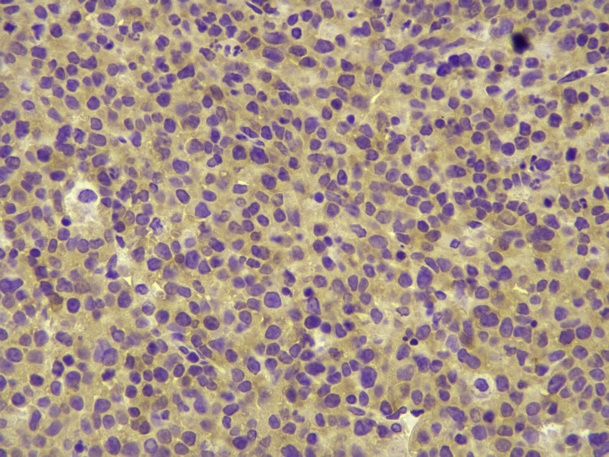

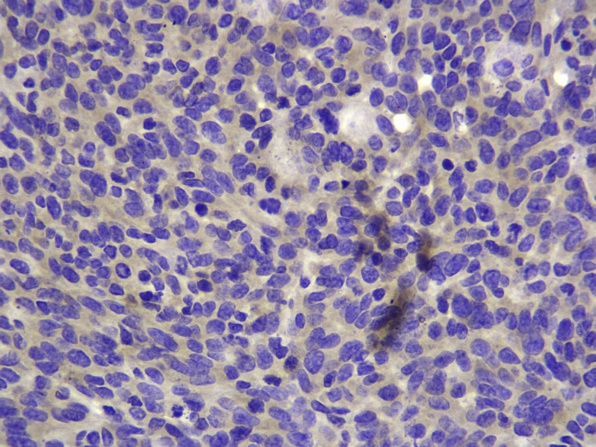


Figure 8 p-STAT3 was detected in HCC827-Gef-control, HCC827-Gef-miR-34a, PC9-Gef-control and PC9-Gef-miR-34a mice by immunohistochemistry（400X）.

HCC827-Gef-control HCC827-Gef-miR-34a


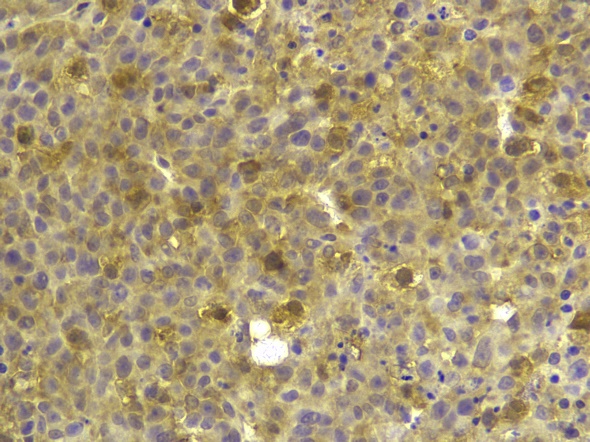

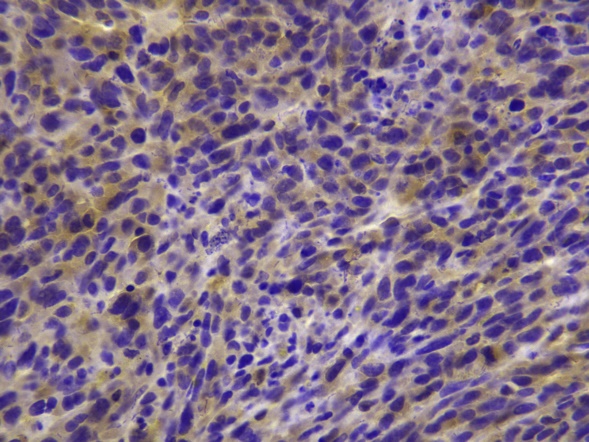


PC9-Gef-control PC9-Gef-miR-34a


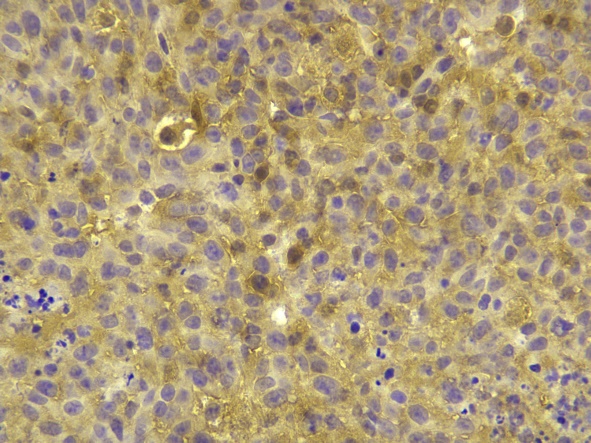

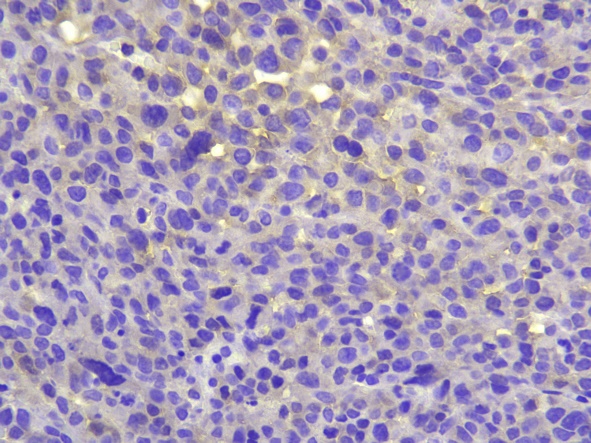

Supplement: Supplementary file 1 — Figure S1 Axl was detected in HCC827‐Gef‐control, HCC827‐Gef‐miR‐34a, PC9‐Gef‐control and PC9‐Gef‐miR‐34a mice by immunohistochemistry(400X). Figure S2 Gas6 was detected in HCC827‐Gef‐control, HCC827‐Gef‐miR‐34a, PC9‐Gef‐control and PC9‐Gef‐miR‐34a mice by immunohistochemistry(400X). Figure S3 AKT was detected in HCC827‐Gef‐control, HCC827‐Gef‐miR‐34a, PC9‐Gef‐control and PC9‐Gef‐miR‐34a mice by immunohistochemistry(400X). Figure S4 p‐AKT was detected in HCC827‐Gef‐control, HCC827‐Gef‐miR‐34a, PC9‐Gef‐control and PC9‐Gef‐miR‐34a mice by immunohistochemistry(400X). Figure S5 ERK was detected in HCC827‐Gef‐control, HCC827‐Gef‐miR‐34a, PC9‐Gef‐control and PC9‐Gef‐miR‐34a mice by immunohistochemistry(400X). Figure S6 p‐ERK was detected in HCC827‐Gef‐control, HCC827‐Gef‐miR‐34a, PC9‐Gef‐control and PC9‐Gef‐miR‐34a mice by immunohistochemistry(400X). Figure S7 STAT3 was detected in HCC827‐Gef‐control, HCC827‐Gef‐miR‐34a, PC9‐Gef‐control and PC9‐Gef‐miR‐34a mice by immunohistochemistry(400X). Figure S8 p‐STAT3 was detected in HCC827‐Gef‐control, HCC827‐Gef‐miR‐34a, PC9‐Gef‐control and PC9‐Gef‐miR‐34a mice by immunohistochemistry(400X). [file TCA-11-156-s001.docx]
